# Rh^II^‐Catalyzed De‐symmetrization of Ethane‐1,2‐dithiol and Propane‐1,3‐dithiol Yields Metallo‐β‐lactamase Inhibitors

**DOI:** 10.1002/cmdc.202100344

**Published:** 2021-08-03

**Authors:** Mikhail Krasavin, Daniil Zhukovsky, Igor Solovyev, Darina Barkhatova, Dmitry Dar'in, Denia Frank, Giada Martinelli, Lilia Weizel, Anna Proschak, Marco Rotter, Jan S. Kramer, Steffen Brunst, Thomas A. Wichelhaus, Ewgenij Proschak

**Affiliations:** ^1^ Institute of Chemistry Saint Petersburg State University 26 Universitetskii prospect Peterhof 198905 Russia; ^2^ Institute of Medical Microbiology and Infection Control University Hospital Frankfurt Paul-Ehrlich-Straße 40 60596 Frankfurt Germany; ^3^ Institute of Pharmaceutical Chemistry Goethe-University Frankfurt Max-von-Laue Str. 9 60438 Frankfurt a.M. Germany

**Keywords:** multiresistant bacteria, metallo β lactamases, thiol inhibitors, Rh(II) catalysis, diazo compounds

## Abstract

Diversity‐oriented synthesis (DOS) is a rich source for novel lead structures in Medicinal Chemistry. In this study, we present a DOS‐compatible method for synthesis of compounds bearing a free thiol moiety. The procedure relies on Rh(II)‐catalyzed coupling of dithiols to diazo building blocks. The synthetized library was probed against metallo‐β‐lactamases (MBLs) NDM‐1 and VIM‐1. Biochemical and biological evaluation led to identification of novel potent MBL inhibitors with antibiotic adjuvant activity.

## Graphical abstract

**Thiols are potent** inhibitors of metallo‐β‐lactamases, major resistance factors in Gram‐negative bacteria. Rh(II)‐based coupling procedure of dithiols to diazo building blocks was adopted for diversity‐oriented synthesis of a library of metallo‐β‐lactamase inhibitors. Most potent compounds displayed nanomolar potency and activity in multiresistent clinical isolates.

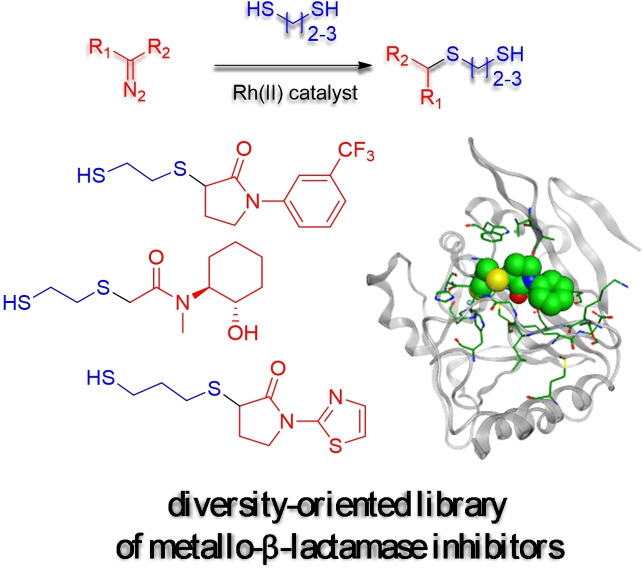

## Introduction

**Figure 1:**
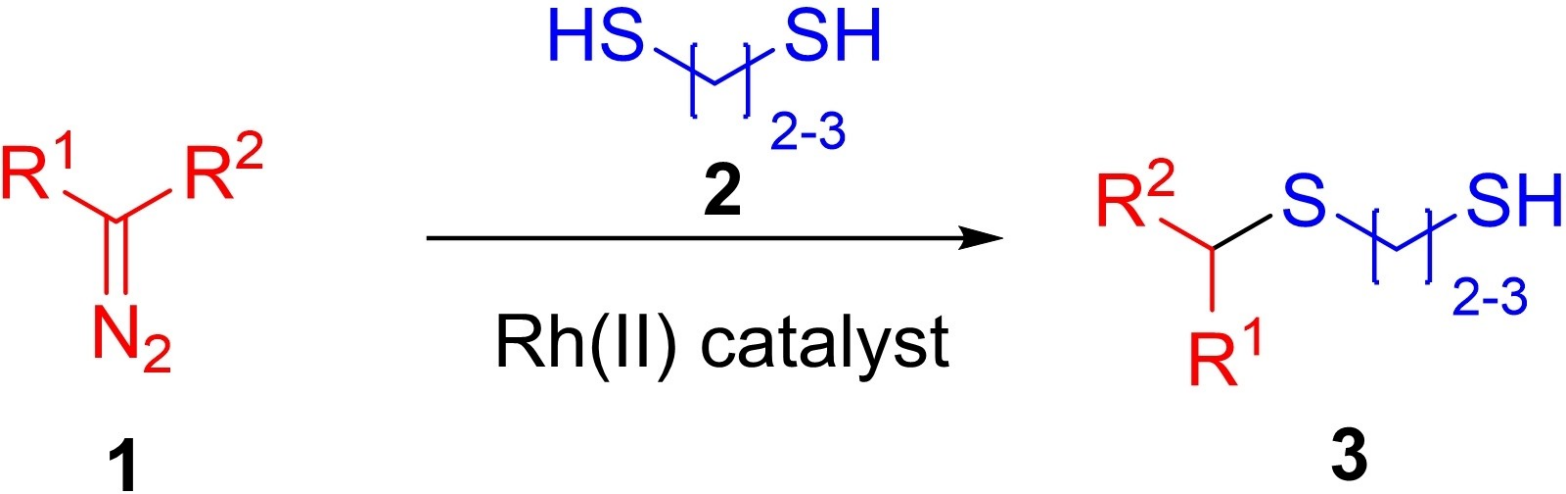
De‐symmetrization of symmetrical aliphatic dithiols **2** *via* Rh(II)‐catalyzed S−H insertion of diazo compounds **1** exploited in this work.

Multiresistant ESKAPE (*Enterococcus faecium, Staphylococcus aureus, Klebsiella pneumoniae, Acinetobacter baumannii, Pseudomonas aeruginosa, and Enterobacter* species) pathogens are a major global threat for human health. Among other resistance factors, β‐lactamases are most prevalent and effectively protect the bacteria against the different kind of β‐lactam antibiotics, including last resort penems and cephalosporins.^[1]^ The β‐lactamase‐mediated mechanism of β‐lactam hydrolysis relies either on nucleophilic serine residue (in serine β‐lactamases, SBLs) or metal ions (in metallo‐ β‐lactamases, MBLs) in the active site of the enzyme. Although in general a β‐lactamase inhibitor does not exhibit antimicrobial activity itself, it prevents the rapid degradation of β‐lactam antibiotics and thereby acts as antibiotic adjuvant.^[2]^ While SBL inhibitors are widely established, agents inhibiting MBLs or both type of β‐lactamases are still under clinical evaluation.^[3]^ Fast evolution of β‐lactamases, caused by high selection pressure make the search for new inhibitors highly urgent. One of the possible MBL inhibition mechanism involves inhibitors possessing a thiol moiety. Thiols bind tightly to the Zn^2+^ ions in the MBL active site.^[4]^ Although a great variety of thiol‐based inhibitors have been developed which reached very significant binding potency *in vitro*,^[5]^ none of them reached clinical evaluation yet. Previously we showed that approved drugs exhibiting free thiol moieties potently inhibit different MBLs *in vitro*.^[6]^ Our efforts to follow the SOSA (selective optimization of side activities) approach^[7]^ to optimize the approved drugs thiorphan and captopril towards efficient MBL inhibitors revealed numerous challenges in the development of thiol‐based MBL inhibitors.^[8]^ Recently, we discovered an efficient Rh(II)‐catalyzed S−H insertion reaction of α‐diazo‐γ‐butyrolactams with a variety of aromatic and aliphatic thiols.^[9]^ Interestingly, the same reaction with ethane‐1,2‐dithiol led to the formation of the mono‐insertion product in good chemical yield. To the best of our knowledge, the latter reaction represents the first example to a general approach to de‐symmetrization of symmetrical aliphatic dithiols **2** using chemistry of diazo compounds **1** (Figure 1). In this article, we describe an application of this novel synthetic approach towards the linking of a thiol moiety to a range of chemically diverse aliphatic scaffolds which led to identification of potent MBL inhibitors among resulting alkylthio‐substituted thiols **3** (Figure 1).

## Results and Discussion

### Synthesis of alkylthio‐substituted aliphatic thiols 3

**Figure 2:**
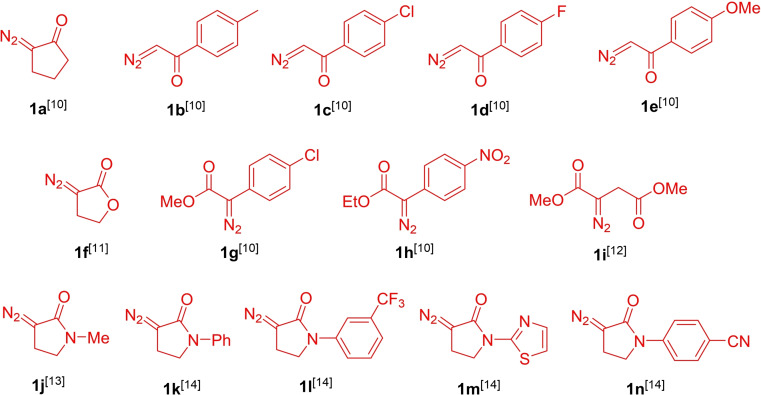
Previously reported diazo compounds **1** **a**–**n** employed in this study.

The arsenal of diazo compounds **1** **a**–**i** selected for this study has been reported previously as prepared *via* the recently developed ‘sulfonyl‐azide‐free’ (SAFE) protocol (*vide infra*).[^10^, ^11^, ^12^] α‐Diazo‐γ‐butyrolactams **1** **j**–**n** were prepared *via* the Danheiser diazo transfer protocol using 4‐nitrobenzene sulfonyl azide as diazo transfer reagent.[^13^, ^14^] It should be noted that all aforementioned diazo compounds are stable to storage except for **1** **j** which undergoes a rapid dimerization^[14]^ and had, therefore, been used immediately^[13]^ in the subsequent Rh(II)‐catalyzed S−H insertion reaction without isolation (Figure 2).

**Scheme 1:**
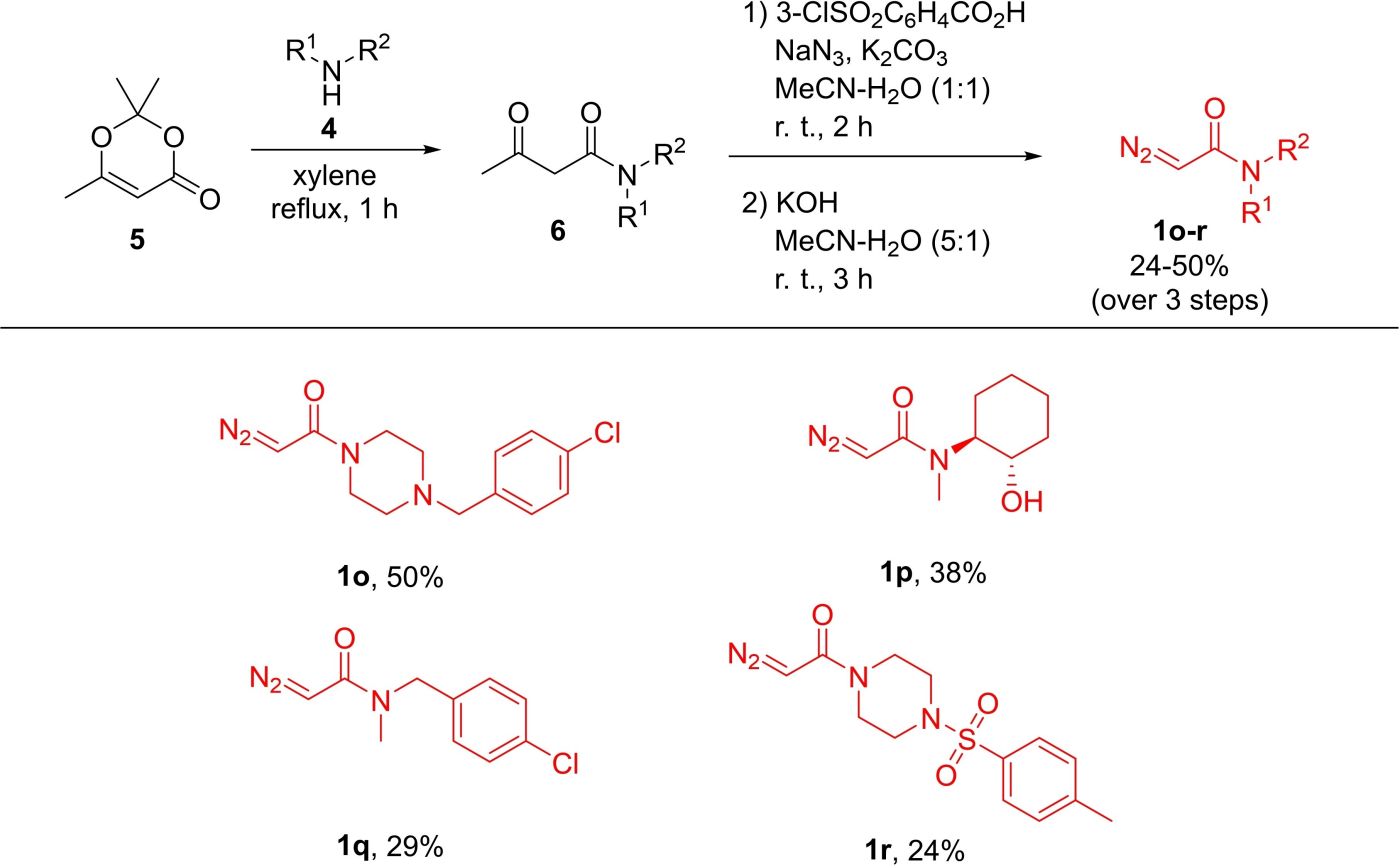
Preparation of α‐diazo acetamides **1** **o**–**r**.

In addition to known α‐diazocarbonyl compounds **1** **a**–**n**, we synthesized a small set of α‐diazo acetamides **1** **o**–**r**. Amines **4** reacted with 2,2,6‐trimethyl‐4*H*‐1,3‐dioxin‐4‐one (**5**) in refluxing xylene which led to ring opening towards α‐acetyl acetamides **6**. The latter, after change of the solvent to acetonitrile, were subjected to the SAFE diazo transfer protocol.^[12]^ After the diazo transfer was complete, brief reaction workup and removal of the acetyl group by treatment with KOH solution in aqueous acetonitrile led to the formation of α‐diazo acetamides **1** **o**–**r**. The latter were isolated by chromatography in modest yields as calculated over 3 chemical steps (Scheme 1).

**Scheme 2:**
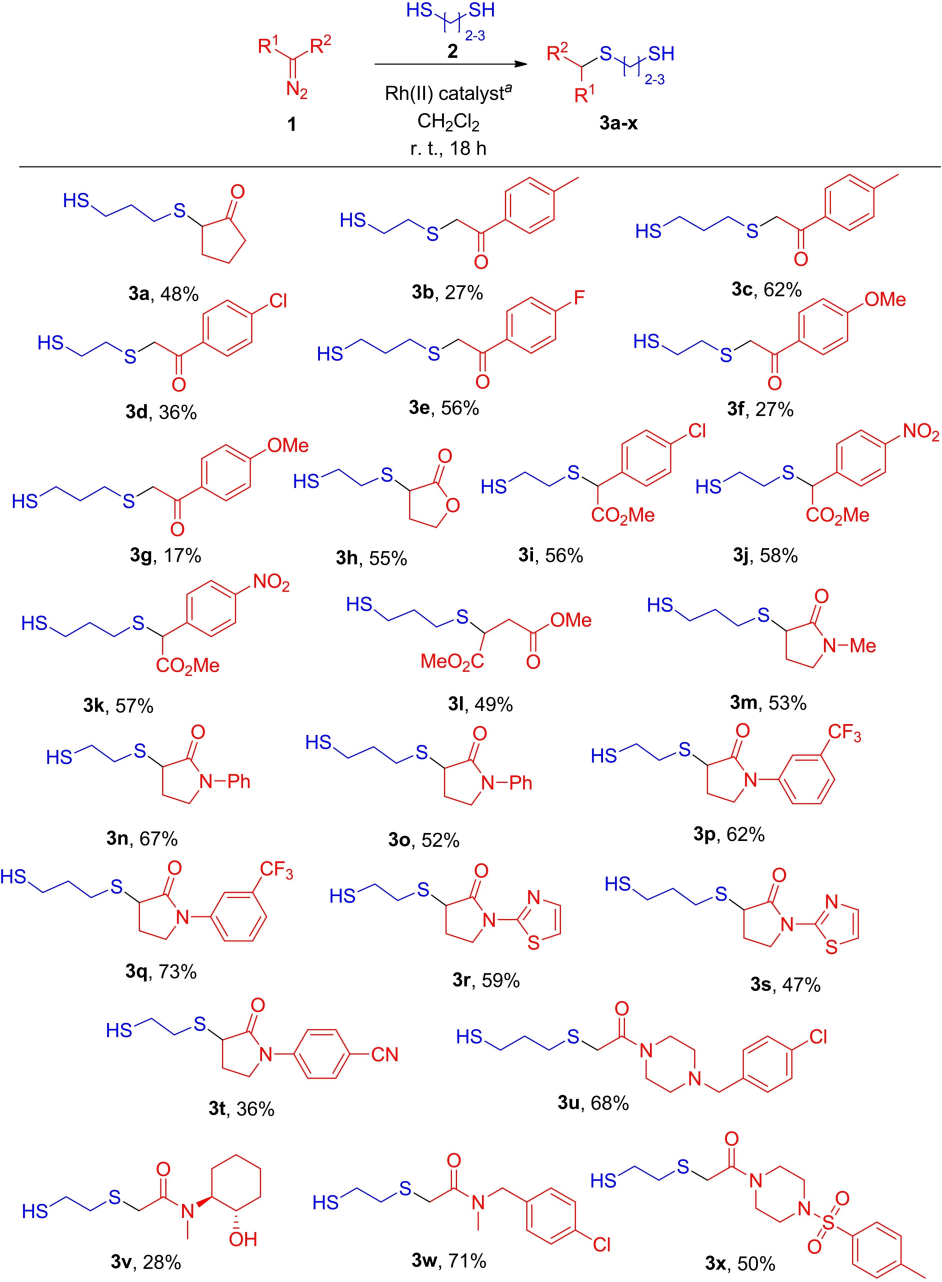
Preparation of alkylthio‐substituted thiols **3** **a**–**x** (^*a*^Rh_2_(OAc)_4_ (1 mol%) for the preparation of compounds **3** **m**–**t**; Rh_2_(esp)_2_ (0.5 mol%)–in all other cases).

With the arsenal of 18 structurally diverse α‐diazocarbonyl compounds **1** **a**–**r** in hand, we proceeded with coupling the respective Rh(II) carbenes to either ethane‐1,2‐dithiol or propane‐1,3‐dithiol, or both, which led to the expected de‐symmetrization of the latter and the formation of alkylthio‐substituted thiols **3** **a**–**x** in modest to good yields (Scheme 2).

### Biochemical evaluation

**Figure 3:**
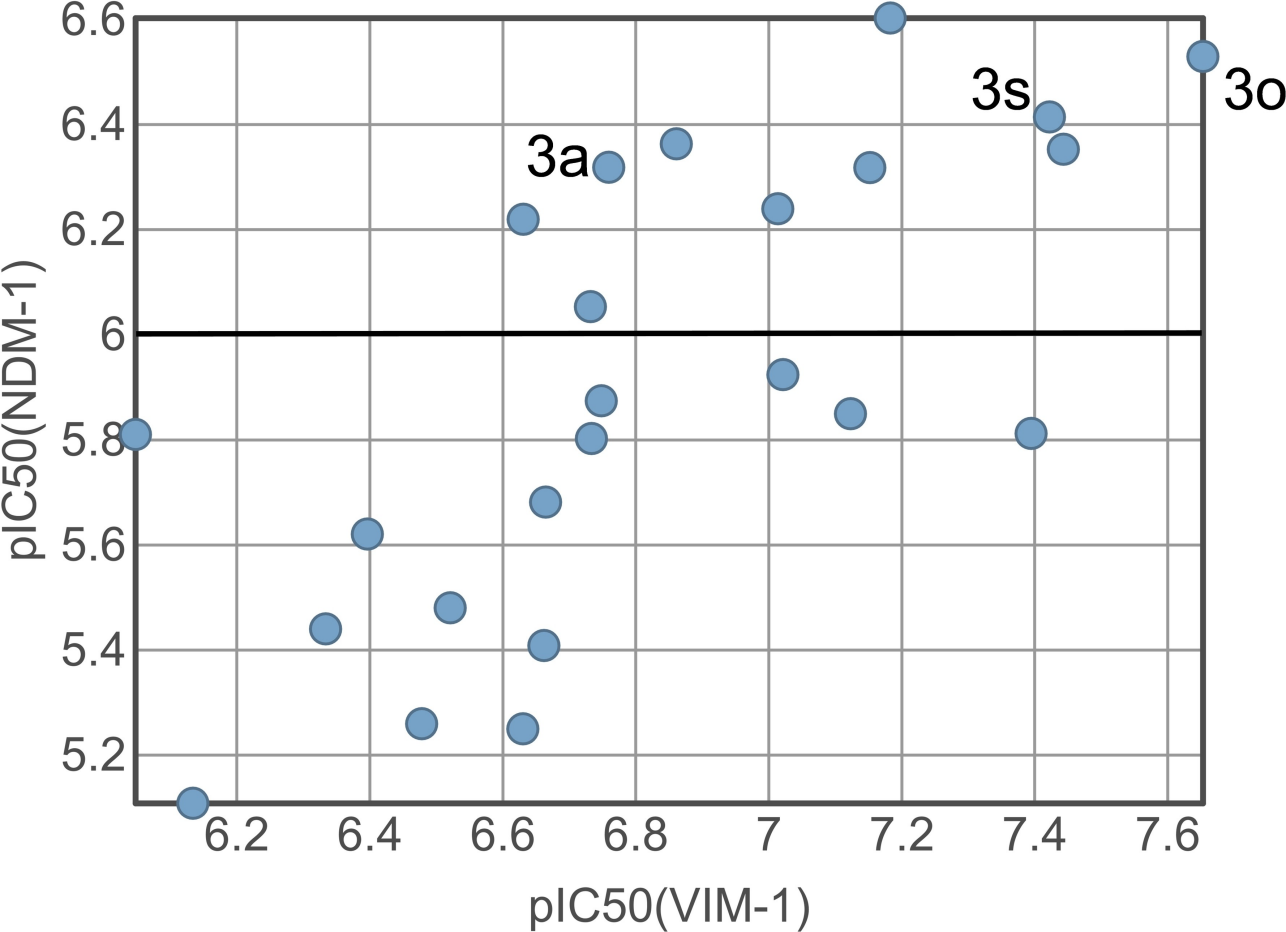
Plot of pIC_50_ values against NDM‐1 and VIM‐1 by compounds **3** **a**–**3** **x**.

In order to investigate the structure‐activity relationships of the dithiol library, we measured the inhibitory activity of **3** **a**–**x** in a fluorescence‐based enzyme activity assay (Table 1). Two relevant β‐lactamase isoforms, NDM‐1 and VIM‐1 were selected for in vitro assays. The fluorogenic substrate fluorocillin^[15]^ was prepared according to literature and its conversion was monitored as described previously.^[6]^ In general, a linear relationship between the potency of **3** **a**–**x** towards both enzymes could be observed, however, inhibitory potency towards VIM‐1 was almost tenfold higher (Figure 3). A very clear preference for the propane‐1,3‐dithiol over ethane‐1,2‐dithiol compounds could be observed from matched molecular pairs. The most potent compound with a balanced inhibitory activity towards both enzymes was the N‐phenyl‐γ‐lactam derivative **3** **o**, which inhibited NDM‐1 with an IC_50_ of 0.3 μM and VIM‐1 with an IC_50_ of 0.02 μM.

**Table 1:** In vitro inhibition of recombinantly expressed and purified MBLs NDM‐1 and VIM‐1 by compounds **3** **a**–**3** **x**.

Compound	IC50 (NDM‐1) [μM]	IC50 (VIM‐1) [μM]
**3** **a**	0.48±0.07	0.17±0.04
**3** **b**	3.91±0.42	0.22±0.03
**3** **c**	0.25±0.06	0.07±0.01
**3** **d**	5.63±1.19	0.23±0.01
**3** **e**	0.60±0.03	0.23±0.05
**3** **f**	1.34±0.32	0.18±0.01
**3** **g**	0.43±0.02	0.14±0.00
**3** **h**	3.63±0.47	0.46±0.03
**3** **i**	0.88±0.16	0.19±0.01
**3** **j**	1.55±0.42	0.90±0.06
**3** **k**	7.79±1.30	0.73±0.05
**3** **l**	0.44±0.11	0.04±0.01
**3** **m**	1.58±0.22	0.18±0.00
**3** **n**	1.41±0.07	0.08±0.00
**3** **o**	0.30±0.04	0.02±0.00
**3** **p**	2.08±0.11	0.22±0.01
**3** **q**	0.48±0.01	0.07±0.01
**3** **r**	1.54±0.03	0.04±0.02
**3** **s**	0.39±0.05	0.04±0.01
**3** **t**	1.19±0.12	0.10±0.02
**3** **u**	3.31±0.12	0.30±0.02
**3** **v**	5.50±0.30	0.33±0.02
**3** **w**	0.58±0.03	0.10±0.01
**3** **x**	2.40±0.13	0.40±0.03

**Figure 4:**
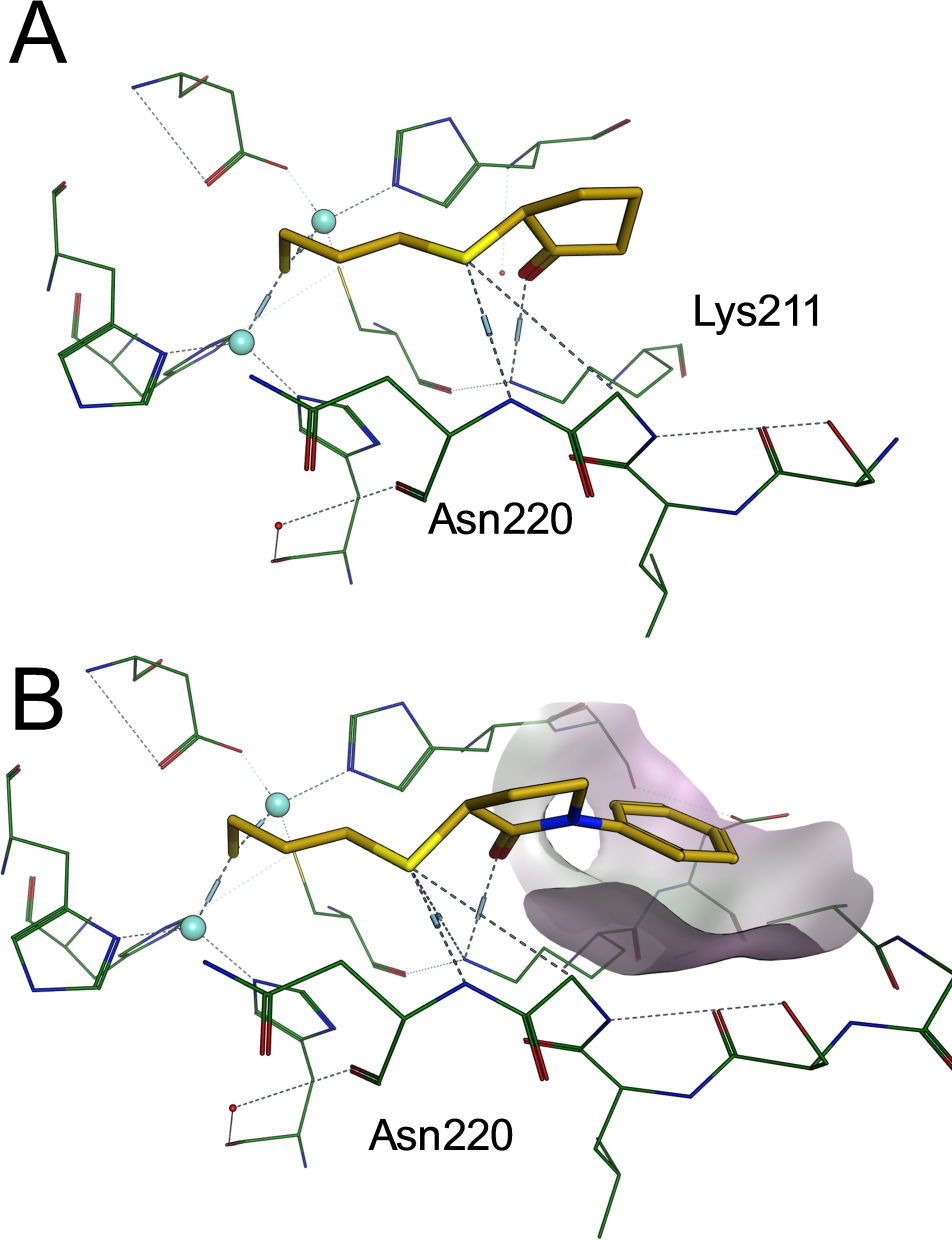
Proposed binding modes of compounds **3** **a** (A) and **3** **o** (B) bound to NDM‐1. Binding modes are generated by molecular docking, the compounds are shown as golden sticks, the protein side chains are displayed as green lines, Zn^2+^ ions are represented as cyan balls. The surface around the phenyl substituent of **3** **o** is coloured by lipophilicity (green: lipophilic, purple: hydrophilic).

In order to rationalize the structure‐activity relationships of the prepared library, molecular docking experiments with the most potent derivative **3** **o** and the most simple analogue **3** **a** were conducted. Therefore, structures of both possible enantiomers of **3** **o** and **3** **a** were docked into the X‐ray structure of NDM‐1 (PDB code 4EXS^[16]^) in complex with a thiol‐containing inhibitor L‐captoptril. The obtained docking mode of **3** **a** revealed that the free thiol group, which was assumed to be negatively charged, is located between the Zn^2+^ ions in the catalytic center. It thereby displaces the polarized water responsible for β‐lactam hydrolysis. Furthermore, the thioether moiety forms a directed hydrogen bond towards backbone NH of Asn220. The carbonyl oxygen of the cyclopentanone moiety forms a hydrogen bond towards the side chain of Lys211. Both interactions towards Asn220 and Lys211 are described to be important for recognition of the carboxylate moiety of β‐lactam.^[17]^ The binding mode explains the preference of the propane‐1,3‐dithiol over ethane‐1,2‐dithiol derivatives, due to the optimal distance of three carbons between the thiol and the thioether groups. (Figure 4)

The docking of the most potent derivative **3** **o** reveals the same preferred interactions as the simplified analogue **3** **a**. (Figure 3B) The N‐phenyl ring reaches out to a flat subpocket which is only partially lipophilic and open to solvent. Due to its open nature, a certain variability can be assumed in this area. This hypothesis fits to the observation that various moieties fit this subpocket without significant loss in activity e. g. N‐phenyl derivative **3** **o** (IC_50_(NDM‐1)=0.3 μM), N‐(3‐CF_3_)‐phenyl derivative **3** **q** (IC_50_(NDM‐1)=0.48 μM), or N‐(2‐thiazolyl) derivative **3** **s** (IC_50_(NDM‐1)=0.39 μM). Notably, only (*S*)‐enantiomers of **3** **a** and **3** **o** were able to display these favourable binding modes while (*R*)‐enantiomers were unable to form all directed interactions in a low‐energy conformation. This observation paves the way for future investigations of the enantioselective synthesis route and subsequent biochemical evaluation of the enantiomers.

**Figure 5:**
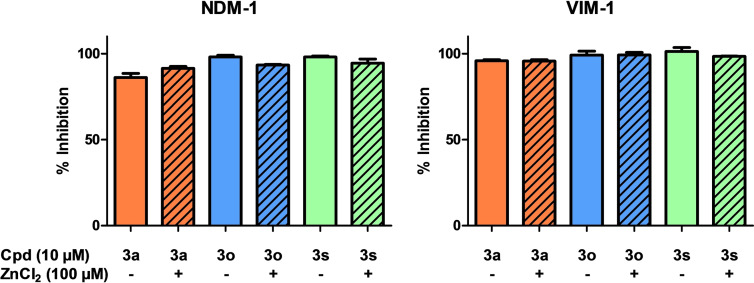
In vitro inhibition of recombinantly expressed and purified MBLs NDM‐1 and VIM‐1 by compounds **3** **a, 3** **o**, and **3** **s** in presence and in absence of 100 μM ZnCl_2_.

For further biological evaluation we concentrated on the compounds with the balanced potency towards both, NDM‐1 and VIM‐1, **3** **o** and **3** **s**. Furthermore, the minimalistic derivative **3** **a** was used for comparison to ensure that the aromatic derivative does not change the mode of action of the compound. Some classes of MBL inhibitors do not directly bind to the active site but act as zinc chelators and withdraw catalytically essential zinc ions.^[18]^ We verified the direct inhibitory mode of action by adding 100 μM ZnCl_2_ to the recombinant MBL in vitro assay. As Figure 5 shows, addition of zinc ions does not impair the inhibitory activity of **3** **a**, **3** **o**, and **3** **s**, suggesting direct inhibition and binding to the active site.

**Figure 6:**
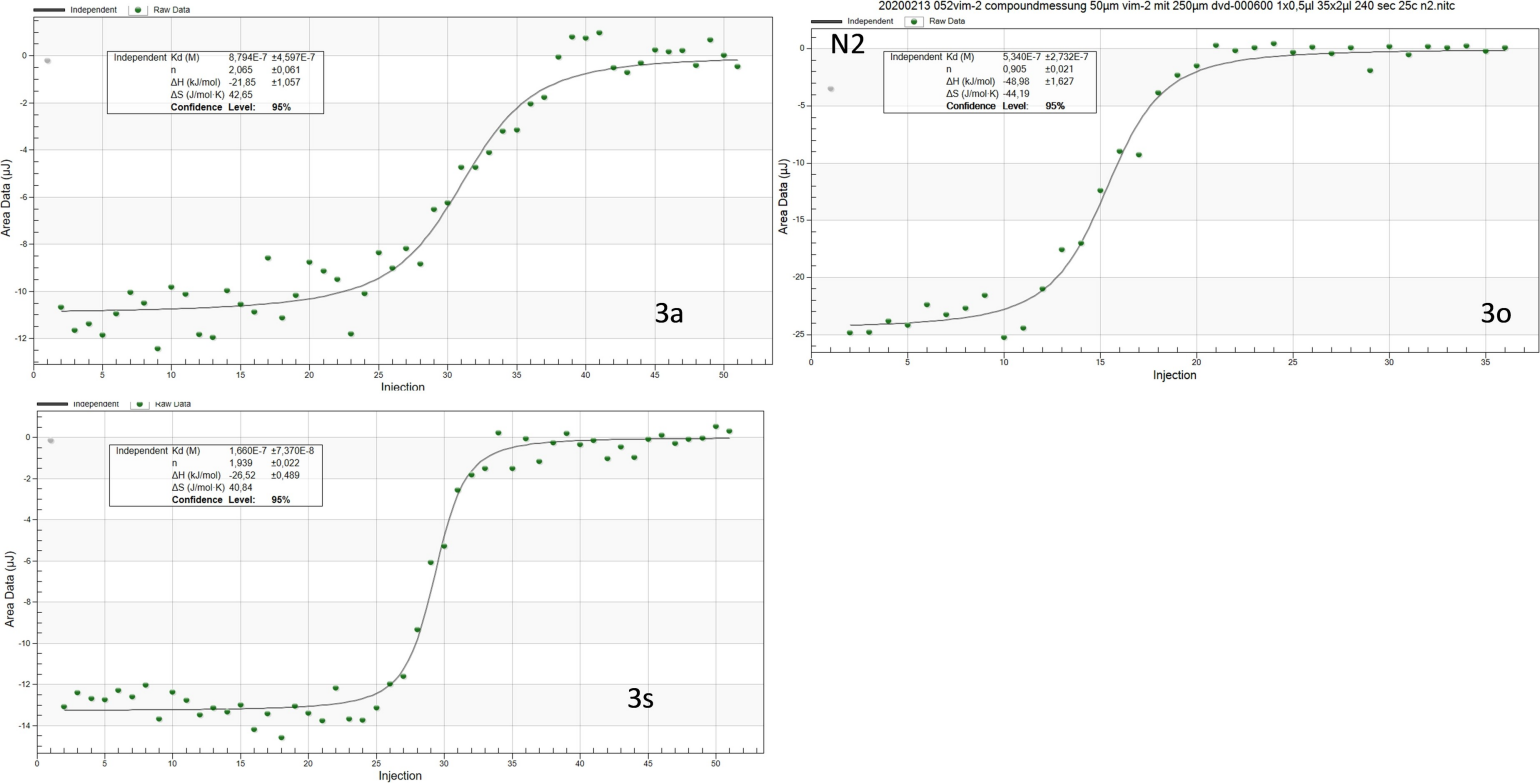
Corrected heat rate of ITC measurements. A) 250 μM **3** **a** into 50 μM VIM‐2; B) 250 μM of **3** **o** into 50 μM VIM‐2; C) 250 μM **3** **s** into 50 μM VIM‐2.

The next step was the investigation of the binding thermodynamics of **3** **a**, **3** **o**, and **3** **s**. For these experiments we used a closely related enzyme VIM‐2 which is an isoform of VIM‐1 and can be recombinantly expressed in very high concentrations, suitable tor isothermal titration calorimetry (ITC) experiments (Figure 6). ITC titration of 250 μM of **3** **a**, **3** **o**, or **3** **s**, respectively, into 50 μM of VIM‐2 revealed potent entropy‐driven binding of all three compounds, with 3a being the weakest (K_d_=0.88 μM). Notably, binding of **3** **o** displays almost double enthalpy ΔH=−49 kJ/mol compared to **3** **a** and **3** **s** (ΔH=−22 kJ/mol and −27 kJ/mol).

The considerable inhibition of purified NDM‐1 and VIM‐1 in vitro suggested that compound **3** **a**, **3** **o**, or **3** **s**, which themselves exhibit no intrinsic antimicrobial activity (Table 2), can potentially restore the activity of imipenem against bacterial isolates producing MBL.

**Table 2:** Intrinsic antimicrobial activity of **3** **a**, **3** **o**, and **3** **s**.

Transformants	MIC in mg/L		
	3a	3o	3 s
*E. coli* NDM‐1 (T2359)	>512	>512	>512
*E. coli* VIM‐1 (T2544)	>512	>512	>512

To investigate this, the MIC of imipenem or combined with various concentrations of compound 3a, 3o, or 3 s was determined against *E. coli* transformants producing NDM‐1 or VIM‐1 (Table 3 and 4). Especially compound 3o and 3 s substantially reduced the MIC of imipenem against NDM‐1 or VIM‐1 producing bacteria up to 8‐fold and 32‐fold, respectively.

**Table 3:** Synergistic antimicrobial activity of imipenem with **3** **a**, **3** **o**, and **3** **s**. against *E. coli* NDM‐1 (T2359).

+ μg/ml	Imipenem MIC in mg/L		
	3a	3o	3 s
0	128	128	128
128	32	16	16
64	64	32	32
32	64	32	64
16	64	128	64
8	128	128	64
4	128	128	128
2	128	128	128

**Table 4:** Synergistic antimicrobial activity of imipenem with **3** **a**, **3** **o**, and **3** **s**. against *E. coli* VIM‐1 (T2544).

+ μg/ml	Imipenem MIC in mg/L		
	3a	3o	3 s
0	32	32	32
128	8	1	2
64	16	2	4
32	16	8	8
16	16	16	16
8	16	16	16
4	32	16	16
2	32	16	32

## Conclusion

In this study we could show that Rh(II)‐catalyzed introduction of dithiols is a highly useful method for diversity‐oriented synthesis of chemical libraries which are intended to contain free sulfhydryl groups. We prepared a diverse library of thiol‐based inhibitors of MBLs and evaluated them in vitro. Biochemical and biological evaluation of the prepared library showed that potent MBL inhibitors with antibiotic adjuvant activity could be generated.

## Experimental Section

### Chemical synthesis

#### General methods

Known diazocarbonyl compounds **1** **a**–**n** were prepared according to literature procedures,[^10^, ^11^, ^12^, ^13^, ^14^] other reagents were obtained from commercial sources and used without any additional purification. Solvents were distilled over suitable drying agents. Mass spectra were recorded with a Bruker Maxis HRMS‐ESI‐qTOF spectrometer (electrospray ionization mode). NMR spectroscopic data were recorded with Bruker Avance 400 spectrometer (400.13 MHz for ^1^H and 100.61 MHz for ^13^C) in CDCl_3_ and were referenced to residual solvent proton peaks (*δ*_H_=7.28) and solvent carbon peaks (*δ*_C_=77.0). Melting points were determined with a Stuart SMP50 instrument in open capillary tubes.

#### Preparation of α‐diazo acetamides 1 o‐r

A mixture of appropriate amine **4** (1 mmol) and 2,2,6‐trimethyl‐4*H*‐1,3‐dioxin‐4‐one (**5**, 1 mmol) in *o*‐xylene (6 mL) was heated at reflux for 1 h and the solvent was removed under reduced pressure. The residue was dissolved in acetonitrile (8 mL) and a mixture of 3‐(chlorosulfonyl)benzoic acid (292 mg, 1.34 mmol), sodium azide (98 mg, 1.5 mmol) and potassium carbonate (276 mg, 2 mmol) in water (8 mL), pre‐stirred over 30 min, was added. The resulting emulsion was stirred for 2 h at room temperature whereupon the diazo transfer was complete. The reaction mixture was extracted with chloroform (2×10 mL). The chloroform solution was separated, dried over anhydrous Na_2_SO_4_, filtered and concentrated to dryness. The residue was dissolved in acetonitrile (20 mL) and was treated with a solution of KOH (140 mg, 5 mmol) in water (4 mL). The resulting mixture was stirred for 3 h at room temperature and extracted with chloroform (2×10 mL). The organic phase was dried over anhydrous Na_2_SO_4_, filtered and the solvent was removed under reduced pressure. The residue was purified by column chromatography using ethyl acetate‐*n*‐hexane 1 : 4 as eluent.

#### 1‐(4‐(4‐Chlorobenzyl)piperazin‐1‐yl)‐2‐diazoethan‐1‐one (1 o)

Yield 139 mg (50 %). Orange semi‐solid. ^1^H NMR (400 MHz, CDCl_3_) δ 7.41–7.30 (m, 2H), 7.30–7.25 (m, 2H), 4.98 (s, 1H), 3.87–2.93 (m, 6H), 2.60–2.11 (m, 4H). ^13^C NMR (101 MHz, CDCl_3_) δ 164.7, 136.2, 133.0, 130.3, 128.5, 62.1, 52.7, 46.4, 43.8. HRMS (ESI/Q‐TOF) m/z: [M+H]^+^ Calcd for C_13_H_16_ClN_4_O 279.1007; Found 279.0999.

#### 2‐Diazo‐*N*‐((1S*,2S*)‐2‐hydroxycyclohexyl)‐*N*‐methylacetamide (1 p)

Yield 75 mg (38 %). Yellow amorphous solid. ^1^H NMR (400 MHz, CDCl_3_) δ 5.06 (s, 1H), 3.67–3.40 (m, 1H), 2.81 (s, 3H), 2.62 (s, 1H), 2.25–2.10 (m, 1H), 1.84–1.68 (m, 3H), 1.59–1.13 (m, 5H). ^13^C NMR (101 MHz, CDCl_3_) δ 167.8, 167.4 (br s), 78.4, 70.0 (br s), 68.4, 60.9, 59.5 (br s), 47.0, 34.8 (br s), 30.6, 29.0 (br s), 28.6, 28.5, 25.0, 24.4, 23.9, 23.8. HRMS (ESI/Q‐TOF) m/z: [M+Na]^+^ Calcd for C_9_H_15_N_3_NaO_2_ 220.1056; Found 220.1060.

#### *N*‐(4‐Chlorobenzyl)‐2‐diazo‐*N*‐methylacetamide (1 q)

Yield 64 mg (29 %). Yellow oil. ^1^H NMR (400 MHz, CDCl_3_) δ 7.33 (d, *J*=8.4 Hz, 2H), 7.20 (d, *J*=8.1 Hz, 2H), 4.99 (s, 1H), 4.51 (s, 2H), 2.86 (s, 3H). ^13^C NMR (101 MHz, CDCl_3_) δ 166.2, 135.7, 133.1, 128.8, 50.9 (br s), 46.5, 34.2. HRMS (ESI/Q‐TOF) m/z: [M+Na]^+^ Calcd for C_10_H_10_ClN_3_NaO 246.0405; Found 246.0408.

#### 2‐Diazo‐1‐(4‐tosylpiperazin‐1‐yl)ethanone (1 r)

Yield 72 mg (24 %). Yellow oil. ^1^H NMR (400 MHz, CDCl_3_) δ 7.70–7.57 (m, 2H), 7.41–7.34 (m, 2H), 4.94 (s, 1H), 3.52 (s, 4H), 3.01 (t, *J*=5.1 Hz, 4H), 2.46 (s, 3H). ^13^C NMR (101 MHz, CDCl_3_) δ 164.8, 144.3, 132.0, 129.9, 127.7, 46.7, 45.9, 43.2 (br s), 21.5. HRMS (ESI/Q‐TOF) m/z: [M+Na]^+^ Calcd for C_13_H_16_N_4_NaO_3_S 331.0835; Found 331.0829.

#### General procedure for the preparation of compounds 3 a‐x

To a vigorously stirred solution of diazo compound **1** (0.5 mmol) and symmetrical dithiol (5 mmol) in dichloromethane (10 mL) an appropriate rhodium(II) catalyst (0.005 mmol of Rh_2_(OAc)_4_ for lactams **3** **m**–**t** and 0.0025 mmol of Rh_2_(esp)_2_ in all other cases) was added. The reaction mixture was stirred at room temperature over 18 h. The volatiles were removed using a rotary evaporator and the desired product was isolated by a column chromatography on silica gel using ethyl acetate–*n*‐hexane 1 : 4 as eluent.

#### 2‐((3‐Mercaptopropyl)thio)cyclopentanone (3 a)

Yield 48 %, 46 mg. Purple amorphous solid. ^1^H NMR (400 MHz, CDCl_3_) δ 3.13–3.04 (m, 1H), 2.82–2.73 (m, 1H), 2.70–2.57 (m, 3H), 2.49–2.39 (m, 1H), 2.31–2.03 (m, 3H), 1.96–1.79 (m, 4H), 1.37 (t, *J*=8.1 Hz, 1H).^13^C NMR (101 MHz, CDCl_3_) δ 213.7, 47.0, 35.9, 32.9, 30.2, 29.2, 23.4, 20.4. HRMS (ESI/Q‐TOF) m/z: [M+Na]^+^ Calcd for C_8_H_14_NaOS_2_ 213.0378; Found 213.0377.

#### 2‐((2‐Mercaptoethyl)thio)‐1‐(*p–*tolyl)ethanone (3 b)

Yield 27 %, 31 mg. Yellow amorphous solid. ^1^H NMR (400 MHz, CDCl_3_, in equilibrium with cyclic semi‐thioacetal) δ 7.88 (d, *J*=8.3 Hz, 2H), 7.29 (d, *J*=8.0 Hz, 2H), 3.81 (s, 2H), 2.85–2.80 (m, 2H), 2.80–2.71 (m, 2H), 2.43 (s, 3H), 1.73–1.66 (m, 1H). ^13^C NMR (101 MHz, CDCl_3_, in equilibrium with cyclic semi‐thioacetal) δ 194.2, 144.5, 132.6, 129.5, 128.9, 36.8, 36.1, 24.1, 21.7. HRMS (ESI/Q‐TOF) m/z: [M+Na]^+^ Calcd for C_11_H_14_NaOS_2_ 249.0378; Found 249.0388.

#### 2‐((3‐Mercaptopropyl)thio)‐1‐(p–tolyl)ethanone (3 c)

Yield 62 %, 74 mg. Yellow amorphous solid. ^1^H NMR (400 MHz, CDCl_3_) δ 7.88 (d, *J*=8.3 Hz, 2H), 7.28 (d, *J*=8.0 Hz, 2H), 3.78 (s, 2H), 2.70 (t, *J*=7.1 Hz, 2H), 2.61 (q, *J*=7.1 Hz, 2H), 2.43 (s, 3H), 1.91 (p, *J*=7.0 Hz, 2H), 1.37 (t, *J*=8.1 Hz, 1H). ^13^C NMR (101 MHz, CDCl_3_) δ 194.2, 144.3, 132.7, 129.4, 128.9, 37.0, 32.7, 30.6, 23.3, 21.7. HRMS (ESI/Q‐TOF) m/z: [M+Na]^+^ Calcd for C_12_H_16_NaOS_2_ 263.0535; Found 263.0540.

#### 1‐(4‐Chlorophenyl)‐2‐((2‐mercaptoethyl)thio)ethanone (3 d)

Yield 36 %, 44 mg. Yellow oil. Thiol: ^1^H NMR (400 MHz, CDCl_3_) δ 7.97–7.83 (m, 2H), 7.49–7.44 (m, 2H), 3.79 (s, 2H), 2.94–2.65 (m, 4H), 1.69 (t, *J*=7.9 Hz, 1H). ^13^C NMR (101 MHz, CDCl_3_) δ 193.2, 140.0, 133.3, 130.2, 129.1, 36.7, 36.1, 24.1. Cyclic semi‐thioacetal: ^1^H NMR (400 MHz, CDCl_3_) δ 7.61 (d, *J*=8.7 Hz, 2H), 7.36 (d, *J*=8.7 Hz, 2H), 4.78 (s, 1H), 3.51–3.41 (m, 1H), 3.12–2.98 (m, 1H), 2.94–2.64 (m, 2H). ^13^C NMR (101 MHz, CDCl_3_) δ 142.1, 134.2, 128.6, 126.5, 75.9, 43.9, 29.0, 27.8. HRMS (ESI/Q‐TOF) m/z: [M+Na]^+^ Calcd for C_10_H_11_ClNaOS_2_ 268.9832; Found 268.9838.

#### 1‐(4‐Fluorophenyl)‐2‐((3‐mercaptopropyl)thio)ethanone (3 e)

Yield 56 %, 68 mg. Yellow oil. ^1^H NMR (400 MHz, CDCl_3_) δ 8.09–7.99 (m, 2H), 7.20–7.13 (m, 2H), 3.78 (s, 2H), 2.72 (t, *J*=7.1 Hz, 2H), 2.63 (q, *J*=7.1 Hz, 2H), 1.92 (p, *J*=7.0 Hz, 2H), 1.38 (t, *J*=8.1 Hz, 1H). ^13^C NMR (101 MHz, CDCl_3_) δ 192.9, 165.9 (d, *J*=255.5 Hz), 131.5 (d, *J*=9.3 Hz), 131.5, 115.9 (d, *J*=22.0), 37.0, 32.6, 30.6, 23.3. HRMS (ESI/Q‐TOF) m/z: [M+Na]^+^ Calcd for C_11_H_13_FNaOS_2_ 267.0284; Found 267.0280.

#### 2‐((2‐Mercaptoethyl)thio)‐1‐(4‐methoxyphenyl)ethanone (3 f)

Yield 27 %, 33 mg. Yellow oil. ^1^H NMR (400 MHz, CDCl_3_) δ 8.00–7.92 (m, 2H), 7.05–6.91 (m, 2H), 3.88 (s, 3H), 3.78 (s, 2H), 2.85–2.79 (m, 2H), 2.78–2.71 (m, 2H), 1.69 (t, *J*=8.0 Hz, 1H). ^13^C NMR (101 MHz, CDCl_3_) δ 193.2, 163.8, 131.1, 128.0, 113.9, 55.5, 36.6, 36.1, 24.2. HRMS (ESI/Q‐TOF) m/z: [M+Na]^+^ Calcd for C_11_H_14_NaO_2_S_2_ 265.0327; Found 265.0326.

#### 2‐((3‐Mercaptopropyl)thio)‐1‐(4‐methoxyphenyl)ethanone (3 g)

Yield 17 %, 22 mg. Yellow oil. ^1^H NMR (400 MHz, CDCl_3_) δ 8.00–7.95 (m, 2H), 6.99–6.93 (m, 2H), 3.89 (s, 3H), 3.76 (s, 2H), 2.71 (t, *J*=7.1 Hz, 2H), 2.62 (q, *J*=7.1 Hz, 2H), 1.92 (p, *J*=7.0 Hz, 2H), 1.37 (t, *J*=8.1 Hz, 1H). ^13^C NMR (101 MHz, CDCl_3_) δ 193.3, 163.8, 131.1, 128.1, 113.9, 55.5, 36.8, 32.7, 30.6, 23.3. HRMS (ESI/Q‐TOF) m/z: [M+Na]^+^ Calcd for C_12_H_16_NaO_2_S_2_ 279.0484; Found 279.0491.

#### 3‐((2‐Mercaptoethyl)thio)dihydrofuran‐2(3*H*)‐one (3 h)

Yield 55 %, 49 mg. Purple oil. ^1^H NMR (400 MHz, CDCl_3_) δ 4.50–4.42 (m, 1H), 4.36 (td, *J*=8.6, 4.1 Hz, 1H), 3.58 (dd, *J*=8.4, 4.3 Hz, 1H), 3.23–3.13 (m, 1H), 2.97–2.88 (m, 1H), 2.87–2.79 (m, 2H), 2.75–2.63 (m, 1H), 2.14 (ddt, *J*=13.6, 7.0, 4.1 Hz, 1H), 1.72 (t, *J*=8.1 Hz, 1H). ^13^C NMR (101 MHz, CDCl_3_) δ 175.2, 66.8, 38.9, 35.2, 29.9, 24.4. HRMS (ESI/Q‐TOF) m/z: [M+Na]^+^ Calcd for C_6_H_10_NaO_2_S_2_ 201.0014; Found 201.0008.

#### Methyl 2‐(4‐chlorophenyl)‐2‐((2‐mercaptoethyl)thio)acetate (3 i)

Yield 56 %, 78 mg. Yellow oil. ^1^H NMR (400 MHz, CDCl_3_) δ 7.45–7.40 (m, 2H), 7.37–7.32 (m, 2H), 4.62 (s, 1H), 3.76 (s, 3H), 2.82–2.74 (m, 2H), 2.72–2.66 (m, 2H), 1.67 (t, *J*=8.1 Hz, 1H). ^13^C NMR (101 MHz, CDCl_3_) δ 170.8, 134.4, 131.5, 129.9, 129.0, 52.9, 51.3, 35.8, 24.3. HRMS (ESI/Q‐TOF) m/z: [M+Na]^+^ Calcd for C_11_H_13_ClNaO_2_S_2_ 298.9938; Found 298.9933.

#### Ethyl 2‐((2‐mercaptoethyl)thio)‐2‐(4‐nitrophenyl)acetate (3 j)

Yield 87 mg, 58 %. Yellow oil. ^1^H NMR (400 MHz, CDCl_3_) δ 8.22 (d, *J*=8.8 Hz, 2H), 7.68 (d, *J*=8.8 Hz, 2H), 4.70 (s, 1H), 4.32–4.15 (m, 2H), 2.89–2.76 (m, 2H), 2.74–2.68 (m, 2H), 1.67 (t, *J*=8.1 Hz, 1H), 1.28 (t, *J*=7.1 Hz, 3H). ^13^C NMR (101 MHz, CDCl_3_) δ 169.6, 147.7, 143.3, 129.6, 123.9, 62.4, 51.4, 36.0, 24.2, 14.1. HRMS (ESI/Q‐TOF) m/z: [M+Na]^+^ Calcd for C_12_H_15_NNaO_4_S_2_ 324.0335; Found 324.0346.

#### Ethyl 2‐((3‐mercaptopropyl)thio)‐2‐(4‐nitrophenyl)acetate (3 k)

Yield 90 mg, 57 %. Yellow oil. ^1^H NMR (400 MHz, CDCl_3_) δ 8.28–8.17 (m, 2H), 7.73–7.65 (m, 2H), 4.64 (s, 1H), 4.30–4.16 (m, 2H), 2.76–2.65 (m, 2H), 2.65–2.56 (m, 2H), 1.93–1.82 (m, 2H), 1.34 (t, *J*=8.1 Hz, 1H), 1.29 (t, *J*=7.1 Hz, 3H). ^13^C NMR (101 MHz, CDCl_3_) δ 169.7, 147.7, 143.5, 129.6, 123.8, 62.3, 51.5, 32.6, 30.3, 23.2, 14.1. HRMS (ESI/Q‐TOF) m/z: [M+Na]^+^ Calcd for C_13_H_17_NNaO_4_S_2_ 338.0491; Found 338.0477.

#### Dimethyl 2‐((3‐mercaptopropyl)thio)succinate (3 l)

Yield 62 mg, 49 %. Yellow oil. ^1^H NMR (400 MHz, CDCl_3_) δ 3.79–3.74 (m, 3H), 3.73–3.63 (m, 4H), 3.01 (ddt, *J*=16.9, 9.5, 1.2 Hz, 1H), 2.86–2.73 (m, 2H), 2.73–2.58 (m, 3H), 1.96–1.85 (m, 2H), 1.37 (t, *J*=8.1 Hz, 1H). ^13^C NMR (101 MHz, CDCl_3_) δ 172.1, 171.0, 52.6, 52.0, 41.4, 36.3, 32.8, 29.8, 23.2. HRMS (ESI/Q‐TOF) m/z: [M+Na]^+^ Calcd for C_9_H_16_NaO_4_S_2_ 275.0382; Found 275.0390.

#### 3‐((3‐Mercaptopropyl)thio)‐1‐methylpyrrolidin‐2‐one (3 m)

Yield 54 mg, 53 %. Yellow oil. ^1^H NMR (400 MHz, CDCl_3_) δ 3.50–3.39 (m, 2H), 3.35–3.25 (m, 1H), 3.02–2.93 (m, 1H), 2.86 (s, 3H), 2.85–2.77 (m, 1H), 2.68–2.61 (m, 2H), 2.46 (dtd, *J*=15.1, 8.5, 6.5 Hz, 1H), 1.98–1.85 (m, 3H), 1.41 (t, *J*=8.1 Hz, 1H). ^13^C NMR (101 MHz, CDCl_3_) δ 173.1, 47.4, 42.7, 33.2, 30.0, 29.4, 26.3, 23.4. HRMS (ESI/Q‐TOF) m/z: [M+Na]^+^ Calcd for C_8_H_15_NNaOS_2_ 228.0487; Found 228.0477.

#### 3‐((2‐Mercaptoethyl)thio)‐1‐phenylpyrrolidin‐2‐one (3 n)

Yield 86 mg, 67 %. Yellow oil. ^1^H NMR (400 MHz, CDCl_3_) δ 7.66–7.60 (m, 2H), 7.44–7.37 (m, 2H), 7.24–7.13 (m, 1H), 3.99 (dt, *J*=9.6, 7.4 Hz, 1H), 3.82 (ddd, *J*=9.6, 8.2, 4.0 Hz, 1H), 3.70 (dd, *J*=8.5, 4.6 Hz, 1H), 3.31–3.21 (m, 1H), 3.00–2.91 (m, 1H), 2.91–2.81 (m, 2H), 2.61 (dtd, *J*=13.4, 8.3, 7.2 Hz, 1H), 2.05 (ddt, *J*=13.5, 7.5, 4.3 Hz, 1H), 1.74 (t, *J*=8.1 Hz, 1H). ^13^C NMR (101 MHz, CDCl_3_) δ 172.1, 139.2, 128.9, 124.9, 120.0, 46.7, 44.1, 35.3, 26.1, 24.7. HRMS (ESI/Q‐TOF) m/z: [M+Na]^+^ Calcd for C_12_H_15_NNaOS_2_ 276.0487; Found 276.0495.

#### 3‐((3‐Mercaptopropyl)thio)‐1‐phenylpyrrolidin‐2‐one (3 o)

Yield 69 mg, 52 %. Yellow amorphous solid. ^1^H NMR (400 MHz, CDCl_3_) δ 7.68–7.56 (m, 2H), 7.45–7.33 (m, 2H), 7.23–7.11 (m, 1H), 3.97 (dt, *J*=9.5, 7.3 Hz, 1H), 3.80 (ddd, *J*=9.6, 8.2, 4.1 Hz, 1H), 3.65 (dd, *J*=8.5, 4.6 Hz, 1H), 3.12–2.98 (m, 1H), 2.88 (dt, *J*=12.9, 7.3 Hz, 1H), 2.67 (dt, *J*=8.2, 7.0 Hz, 2H), 2.63–2.54 (m, 1H), 2.11–1.92 (m, 3H), 1.43 (t, *J*=8.1 Hz, 1H). ^13^C NMR (101 MHz, CDCl_3_) δ 172.2, 139.3, 128.9, 124.8, 120.0, 46.7, 44.2, 33.2, 29.6, 26.1, 23.5. HRMS (ESI/Q‐TOF) m/z: [M+Na]^+^ Calcd for C_13_H_17_NNaOS_2_ 268.0824; Found 268.0830.

#### 3‐((2‐Mercaptoethyl)thio)‐1‐(3‐(trifluoromethyl)phenyl)pyrrolidin‐2‐one (3 p)

Yield 100 mg, 62 %. Yellow oil. ^1^H NMR (400 MHz, CDCl_3_) δ 7.98–7.85 (m, 2H), 7.56–7.48 (m, 1H), 7.46–7.41 (m, 1H), 4.02 (dt, *J*=9.5, 7.4 Hz, 1H), 3.84 (ddd, *J*=9.5, 8.2, 3.9 Hz, 1H), 3.72 (dd, *J*=8.5, 4.5 Hz, 1H), 3.32–3.19 (m, 1H), 3.01–2.91 (m, 1H), 2.90–2.79 (m, 2H), 2.64 (dtd, *J*=13.5, 8.4, 7.3 Hz, 1H), 2.08 (ddt, *J*=13.6, 7.6, 4.2 Hz, 1H), 1.75 (t, *J*=8.1 Hz, 1H). ^13^C NMR (101 MHz, CDCl_3_) δ 172.4, 139.7, 131.3 (q, *J*=32.4 Hz), 129.5, 123.9 (q, *J*=272.5 Hz), 122.8, 121.3 (q, *J*=3.8 Hz), 116.3 (q, *J*=3.8 Hz), 46.5, 43.9, 35.3, 25.9, 24.6. HRMS (ESI/Q‐TOF) m/z: [M+Na]^+^ Calcd for C_13_H_14_F_3_NNaOS_2_ 322.0542; Found 322.0532.

#### 3‐((3‐Mercaptopropyl)thio)‐1‐(3‐(trifluoromethyl)phenyl)pyrrolidin‐2‐one (3 q)

Yield 122 mg, 73 %. Yellow oil. ^1^H NMR (400 MHz, CDCl_3_) δ 7.99–7.81 (m, 2H), 7.54–7.48 (m, 1H), 7.45–7.41 (m, 1H), 4.00 (dt, *J*=9.5, 7.4 Hz, 1H), 3.83 (ddd, *J*=9.4, 8.2, 4.1 Hz, 1H), 3.66 (dd, *J*=8.5, 4.6 Hz, 1H), 3.10–2.98 (m, 1H), 2.92–2.83 (m, 1H), 2.71–2.55 (m, 3H), 2.08 (ddt, *J*=13.5, 7.5, 4.3 Hz, 1H), 2.03–1.90 (m, 2H), 1.42 (t, *J*=8.1 Hz, 1H). ^13^C NMR (101 MHz, CDCl_3_) δ 172.5, 139.8, 131.3 (q, *J*=32.4 Hz), 129.5, 123.9 (q, *J*=272.5 Hz), 122.8, 121.2 (q, *J*=3.8 Hz), 116.3 (q, *J*=3.9 Hz), 46.5, 44.1, 33.1, 29.6, 25.9, 23.4. HRMS (ESI/Q‐TOF) m/z: [M+Na]^+^ Calcd for C_14_H_16_F_3_NNaOS_2_ 358.0518; Found 358.0529.

#### 3‐((2‐Mercaptoethyl)thio)‐1‐(thiazol‐2‐yl)pyrrolidin‐2‐one (3 r)

Yield 77 mg, 59 %. Yellow oil. ^1^H NMR (400 MHz, CDCl_3_) δ 7.51 (d, *J*=3.6 Hz, 1H), 7.06 (d, *J*=3.6 Hz, 1H), 4.29–4.12 (m, 2H), 3.98–3.69 (m, 1H), 3.30–3.14 (m, 1H), 3.05–2.91 (m, 1H), 2.91–2.79 (m, 2H), 2.73–2.65 (m, 1H), 2.31–2.00 (m, 1H), 1.74 (t, *J*=8.1 Hz, 1H). ^13^C NMR (101 MHz, CDCl_3_) δ 171.5, 157.6, 137.8, 114.2, 46.0, 43.1, 35.4, 26.2, 24.6. HRMS (ESI/Q‐TOF) m/z: [M+Na]^+^ Calcd for C_9_H_12_N_2_NaOS_3_ 261.0185; Found 261.0184.

#### 3‐((3‐Mercaptopropyl)thio)‐1‐(thiazol‐2‐yl)pyrrolidin‐2‐one (3 s)

Yield 64 mg, 47 %. Yellow oil. ^1^H NMR (400 MHz, CDCl_3_) δ 7.50 (d, *J*=3.5 Hz, 1H), 7.05 (d, *J*=3.5 Hz, 1H), 4.25–4.12 (m, 2H), 3.73 (dd, *J*=8.6, 4.6 Hz, 1H), 3.04 (ddd, *J*=12.9, 7.5, 6.3 Hz, 1H), 2.88 (dt, *J*=12.8, 7.3 Hz, 1H), 2.73–2.57 (m, 3H), 2.12 (ddt, *J*=13.7, 7.2, 4.7 Hz, 1H), 2.06–1.89 (m, 2H), 1.41 (t, *J*=8.1 Hz, 1H). ^13^C NMR (101 MHz, CDCl_3_) δ 171.6, 157.7, 137.7, 114.2, 46.1, 43.2, 33.0, 29.8, 26.3, 23.4. HRMS (ESI/Q‐TOF) m/z: [M+Na]^+^ Calcd for C_10_H_14_N_2_NaOS_3_ 297.0160; Found 297.0171.

#### 4‐(3‐((2‐Mercaptoethyl)thio)‐2‐oxopyrrolidin‐1‐yl)benzonitrile (3 t)

Yield 50 mg, 36 %. Yellow oil. ^1^H NMR (400 MHz, CDCl_3_) δ 7.84–7.77 (m, 2H), 7.72–7.65 (m, 2H), 4.00 (dt, *J*=9.6, 7.5 Hz, 1H), 3.84 (ddd, *J*=9.5, 8.3, 3.8 Hz, 1H), 3.72 (dd, *J*=8.4, 4.3 Hz, 1H), 3.28–3.17 (m, 1H), 2.97–2.90 (m, 1H), 2.90–2.82 (m, 2H), 2.64 (dq, *J*=13.6, 8.2 Hz, 1H), 2.09 (ddt, *J*=13.6, 7.7, 4.0 Hz, 1H), 1.74 (t, *J*=8.1 Hz, 1H). ^13^C NMR (101 MHz, CDCl_3_) δ 172.8, 143.0, 133.1, 119.4, 118.7, 107.6, 46.3, 44.0, 35.4, 25.7, 24.6. HRMS (ESI/Q‐TOF) m/z: [M+Na]^+^ Calcd for C_13_H_14_N_2_NaOS_2_ 301.0440; Found 301.0431.

#### 1‐(4‐(4‐Chlorobenzyl)piperazin‐1‐yl)‐2‐((3‐mercaptopropyl)thio)ethan‐1‐one (3 u)

Yield 122 mg, 68 %. Yellow oil. ^1^H NMR (400 MHz, CDCl_3_) δ 7.31 (d, *J*=8.3 Hz, 2H), 7.27 (d, *J*=8.5 Hz, 2H), 3.63 (t, *J*=5.1 Hz, 2H), 3.54–3.45 (m, 4H), 3.30 (s, 2H), 2.77 (t, *J*=7.1 Hz, 2H), 2.68–2.60 (m, 2H), 2.47 (t, *J*=5.0 Hz, 2H), 2.43 (t, *J*=5.2 Hz, 2H), 1.94 (p, *J*=7.0 Hz, 2H), 1.39 (t, *J*=8.0 Hz, 1H). ^13^C NMR (101 MHz, CDCl_3_) δ 167.6, 136.3, 133.0, 130.3, 128.5, 62.0, 53.0, 52.7, 46.4, 41.8, 33.2, 32.9, 30.5, 23.3. HRMS (ESI/Q‐TOF) m/z: [M+H]^+^ Calcd for C_16_H_24_ClN_2_OS_2_ 359.1013; Found 359.1008.

#### *N*‐((1S*,2S*)‐2‐Hydroxycyclohexyl)‐2‐((2‐mercaptoethyl)thio)‐N‐methylacetamide (3 v)

Yield 28 %, 37 mg. Yellow oil. ^1^H NMR (400 MHz, CDCl_3_) δ 4.36–4.22 (m, 0.5H), 3.71–3.41 (m, 2H), 3.41–3.29 (m, 1.5H), 2.99 (s, 1.5H), 2.95–2.83 (m, 3H), 2.83–2.74 (m, 2H), 2.19–2.06 (m, 1H), 1.92–1.67 (m, 4H), 1.63–1.08 (m, 5H), 1.02–0.78 (m, 0.5H). ^13^C NMR (101 MHz, CDCl_3_) δ 170.8, 170.2, 70.5, 69.6, 63.8, 59.3, 36.1, 35.9, 35.2, 34.6, 33.8, 33.5, 30.3, 29.4, 28.5, 27.3, 25.1, 25.0, 24.5, 24.4, 24.3, 24.3, 24.3. HRMS (ESI/Q‐TOF) m/z: [M+H]^+^ Calcd for C_11_H_22_NO_2_S_2_ 264.1086; Found 264.1095.

#### *N*‐(4‐Chlorobenzyl)‐2‐((2‐mercaptoethyl)thio)‐*N*‐methylacetamide (3 w)

Yield 71 %, 103 mg. Yellow oil. ^1^H NMR (400 MHz, CDCl_3_) δ 7.38–7.27 (m, 2H), 7.24–7.12 (m, 2H), 4.62–4.51 (m, 2H), 3.39–3.30 (m, 2H), 3.00 (s, 3H), 2.96–2.89 (m, 2H), 2.85–2.75 (m, 2H), 1.74–1.68 (m, 1H). ^13^C NMR (101 MHz, CDCl_3_) δ 169.5, 169.2, 135.5, 134.8, 133.7, 133.3, 129.3, 129.2, 128.8, 127.9, 53.3, 50.5, 36.2, 36.0, 35.6, 34.1, 33.0, 32.9, 24.3. HRMS (ESI/Q‐TOF) m/z: [M+H]^+^ Calcd for C_12_H_17_ClNOS_2_ 290.0435; Found 290.0443.

#### 2‐((2‐Mercaptoethyl)thio)‐1‐(4‐tosylpiperazin‐1‐yl)ethanone (3 x)

Yield 50 %, 94 mg. White solid, mp 100.5‐101.4 °C. ^1^H NMR (400 MHz, CDCl_3_) δ 7.69–7.58 (m, 2H), 7.38–7.33 (m, 2H), 3.70 (t, *J*=5.1 Hz, 2H), 3.58 (t, *J*=5.0 Hz, 2H), 3.26 (s, 2H), 3.09 (t, *J*=5.0 Hz, 2H), 3.00 (t, *J*=5.1 Hz, 2H), 2.84–2.75 (m, 2H), 2.72–2.63 (m, 2H), 2.45 (s, 3H), 1.56 (t, *J*=8.0 Hz, 1H). ^13^C NMR (101 MHz, CDCl_3_) δ 167.6, 144.2, 132.3, 129.9, 127.8, 46.0, 45.9, 45.8, 41.1, 36.1, 32.8, 24.2, 21.6. HRMS (ESI/Q‐TOF) m/z: [M+H]^+^ Calcd for C_15_H_23_N_2_O_3_S_3_ 375.0865; Found 375.0882.

### Biological evaluation

#### Inhibition of VIM‐1 and NDM‐1 metallo‐β ‐lactamases

Activity assays for MBLs were performed at room temperature in black polystyrol 96well plates (Corning, Corning, NY, USA) using dicefalotinodifluorofluorescein (Fluorocillin) as a substrate. Proteins were diluted in assay buffer (HEPES 50 mM, pH 7.5 containing 0.01 %Triton X‐100), with final protein concentrations of NDM‐1: 3 nM, or VIM‐1: 4 nM. Samples were supplemented with an equimolar amount of ZnCl_2_. An amount of 1 μL of compounds **3** **a**–**3** **x** at different concentrations was incubated with 89 μL of enzyme in assay buffer. After an incubation period of 30 min at room temperature, 10 μL of Fluorocillin substrate was added to yield the final assay volume of 100 μL. Final concentration of DMSO was 1 %. The fluorescence emitted by the fluorescent product difluorofluorescein was monitored every 45 s for 30 cycles using a Tecan fluorescent plate reader (Infinite 200; excitation at 495 nm and emission at 525 nm) and was compared with a standard curve. The rate of the enzymatic reaction was obtained by dividing the quantity of the fluorescent product (RFU) by time (min). Negative controls were measured in the absence of enzyme, whereas the positive controls were measured in the presence of enzyme and in the absence of inhibitors. The inhibitory effect of each substance was measured in triplicate in three independent experiments. IC_50_ values were calculated using data obtained from measurements with at least eight different inhibitor concentrations, applying a sigmoidal dose–response (variable slope with four parameters) equation using GraphPad Prism 5 (GraphPad Software, La Jolla, CA, USA) software. In order to investigate the inhibitory activity of **3** **a, 3** **o**, or **3** **s** in the presence of a high Zn^2+^ concentration, assay buffer containing an additional 100 μM ZnCl2 was used. The assay was performed as described above with NDM‐1 or VIM‐1 and 10 μM of **3** **a, 3** **o**, or **3** **s**.

#### K_d_ determination using isothermal titration calorimetry

VIM‐2 expression and purification was published elsewhere.^[19]^ Before the measurement the VIM‐2 protein was dialyzed against 500 times the volume of buffer (50 mM Tris, 500 mM NaCl, 5 % (v/v) glycerol with a pH=8) using a dialysis membrane with a 3.5 kDa MWCO. For the measurements the compounds **3** **a**, **3** **o**, or **3** **s**, respectively, were diluted using the dialyses buffer to a final concentration of 250 μM containing a final DMSO concentration of 1 %. The VIM‐2 protein was diluted to a final concentration of 50 μM, supplemented with 1 % DMSO using the dialysis buffer and pure DMSO. For the control measurements dialysis buffer was supplemented with pure DMSO to a final concentration of 1 %. The measurements were performed using an “Affinity ITC” (TA‐Instruments) in reversed mode, with a stir rate of 75 rpm and a temperature of 25 °C. The VIM‐2 was placed in the cell and the respective compound in the syringe. For blank measurements either the protein or compound dilution were exchanged with buffer containing 1 % DMSO. For the measurements of **3** **a** and **3** **s** one time 0.5 μl was injected followed by 50 injections of 2 μl with a spacing of 240 s. **3** **o** was measured with one injection of 0.5 μl followed by 35 injections of 2 μl with a spacing of 240 s. The data were analyzed using the “NanoAnalyze Data Analysis” software (version 3.10.0; TA‐Instruments).

#### Minimal inhibitory concentration determination

Minimal inhibitory concentrations (MICs) of imipenem monohydrate (Sigma‐Aldrich)±compound **3** **a**, **3** **o**, or **3** **s** against transformed *E. coli* strains producing NDM‐1 or VIM‐1 were determined according to microdilution method established by Clinical and Laboratory Standards Institute (CLSI).^20^ The checkerboard assay was performed to test for synergy in vitro. The microtiter‐plates were set up with serial doubling dilutions of compound **3** **a**, **3** **o**, or **3** **s** (2‐128 mg/L) and imipenem (0.125‐128 mg/L).

#### Molecular modeling

Docking was performed using MOE2019.0102 (Chemical Computing Group, Montreal, Canada). X‐ray structure of NDM‐1 (PDB code 4EXS^16^) was downloaded from PDB and prepared using QuickPrep routine. Co‐crystallized ligand captopril was selected to define the binding site. Induced‐fit docking was employed to dock both enantiomers of **3** **a** and **3** **o**, respectively. For initial placement, template “CHCH_2_S^−^” was used, while rescoring of the obtained conformations was performed by GBVI/WSA dG scoring function to generate 5 low‐energy docking poses, which were inspected manually. Poses with the highest score were used for generation of Figure 4.

## Conflict of interest

The authors declare no conflict of interest.
